# Functional interaction between *Env *oncogene from Jaagsiekte sheep retrovirus and tumor suppressor Sprouty2

**DOI:** 10.1186/1742-4690-7-62

**Published:** 2010-08-02

**Authors:** Ebenezer Chitra, Yi-Wen Lin, Fabian Davamani, Kuang-Nan Hsiao, Charles Sia, Shih-Yang Hsieh, Olivia L Wei, Jen-Hao Chen, Yen-Hung Chow

**Affiliations:** 1 Vaccine R&D Center, National Health Research Institutes, 35, Keyan Road, Zhunan, Miaoli County 350, Taiwan; 2 Current Address: School of Medical Sciences, Division of Human Biology, International Medical University, Kuala Lumpur, Malaysia; 3 The Graduate Division of Biological and Biomedical Sciences (GDBBS), Emory University, Atlanta, Georgia, GA 30322, USA; 4 Department of Dentistry, Kaohsiung Municipal Hsiaokang Hospital, School of Dentistry, College of Dental Medicine, Kaohsiung Medical University, Kaohsiung, Taiwan; 5 Graduate Program of Biotechnology in Medicine, Institute of Molecular Medicine, National Tsing Hua University, Hsinchu 300, Taiwan

## Abstract

**Background:**

Jaagsiekte sheep retrovirus (JSRV) is a type D retrovirus capable of transforming target cells *in vitro *and *in vivo*. The Envelope *(Env) *gene from JSRV and from related retroviruses can induce oncogenic transformation, although the detailed mechanism is yet to be clearly understood. Host cell factors are envisaged to play a critical determining role in the regulation of *Env*-mediated cell transformation.

**Results:**

JSRV *Env*-mediated transformation of a lung adenocarcinoma cell line induced rapid proliferation, anchorage-independent growth and tumor formation, but completely abrogated the migration ability. An analysis of the signaling scenario in the transformed cells suggested the involvement of the ERK pathway regulated by Sprouty2 in cell migration, and the PI3K-Akt and STAT3 pathways in proliferation and anchorage-independence. On the other hand, in a normal lung epithelial cell line, *Env*-mediated transformation only decreased the migration potential while the other functions remained unaltered. We observed that *Env *induced the expression of a tumor suppressor, Sprouty2, suggesting a correlation between *Env*-effect and Sprouty2 expression. Overexpression of Sprouty2 *per se *not only decreased the migratory potential and tumor formation potential of the target cells but also made them resistant to subsequent *Env*-mediated transformation. On the other hand, over expression of the functional mutants of Sprouty2 had no inhibitory effect, confirming the role of Sprouty2 as a tumor suppressor.

**Conclusions:**

Our studies demonstrate that *Env *and Sprouty2 have a functional relationship, probably through shared signaling network. Sprouty2 functions as a tumor suppressor regulating oncogenic transformation of cells, and it therefore has the potential to be exploited as a therapeutic anti-cancer agent.

## Background

The Envelope proteins of many retroviruses have been identified to be directly involved in oncogenic transformation of cells leading to the evolution of a new paradigm. Friend Spleen Focus Forming Virus (SFFV) was the first virus to be identified to be linked to oncogenesis induced by a retroviral Env protein [1]. Tumor formation by SFFV was reported to involve the mitogen-activated protein kinase (MAPK) and the phosphatidylinositol 3-kinase (PI3K) pathways, with a number of host factors governing the susceptibility to tumor formation [1]. Structural proteins of Avian Hemangioma Virus (AHV) and Mouse Mammary Tumor Virus (MMTV) have also been shown to be involved in oncogenic transformation [1]. *Env *genes from Jaagsiekte sheep retrovirus (JSRV) and Enzootic Nasal Tumor Virus (ENTV) are both known to act as oncogenes. They can transform cell lines *in vitro*, using similar set of signaling pathways involving the MAPK and PI3K, and when expressed *in vivo *they can induce tumors in animals [2-4]. Detailed investigation of the retroviral *Env *genes could reveal the underlying mechanisms and signaling pathways implicated in oncogenic transformation.

JSRV is an acutely transforming betaretrovirus that induces contagious pulmonary adenocarcinoma in sheep [5] which resembles a subtype of human adenocarcinoma [6]. The *Env *oncogene of JSRV is capable of transforming target cells *in vivo *as well as *in vitro*, acting through the PI3K/Akt and MAPK signaling pathways [3,7-10]. The JSRV Envelope protein harbors a putative binding site for the p85 regulatory subunit of PI3K in its cytoplasmic tail [11], and the amino acid Y590 present at this site is envisaged to play a crucial role in tumorigenesis [12]; mutation of this amino acid has been reported to reduce the transformation efficiency of Envelope [13,14]. The surface domain of JSRV Envelope protein is capable of activating an independent signaling pathway leading to the transformation of target cells [15]. Induction of the PI3K/Akt pathway is considered essential for *Env*-mediated cellular transformation [13]. However, in some cell types, *Env*-mediated transformation induced the MAPK pathway [8], suggesting that both the PI3K and MAPK pathways can be modulated by *Env*. Development of lung tumors has been reported by lung-specific expression of *Env *gene in transgenic [16] or normal mice [3], confirming its role as an oncogene.

Cell growth control networks involve oncoprotein- and tumor suppressor protein-regulated signaling pathways with increasingly diverse functions and complex interactions for each set of proteins. While some oncoprotein-tumor suppressor pairs like Mdm2 and p53 [17], mixed lineage leukemia protein and menin [18], MSP58 and PTEN [19] are capable of direct physical interaction, other cryptic indirect interactions are yet to be unraveled. This study focuses on the functional interaction between the *Env *oncogene of Jaagsiekte sheep retrovirus (JSRV) and the tumor suppressor, human Sprouty2.

The Sprouty family comprises of non-autonomous signaling proteins that function in feedback circuits involving the Ras/MAP kinase pathway [20,21] and act as tumor suppressors. Sprouty was first discovered in Drosophila [22], and later its isoforms were identified in many organisms. Human Sprouty2 is a 35 kDa polypeptide known to associate with a wide range of signaling molecules like c-Cbl [23], human Seven in Absentia homolog 2 (SIAH2) [24], protein phosphatase 2A (PP2A) [25] and the adaptor protein, CrkL [26] by means of its key tyrosine residue Y55, which is tyrosine phosphorylated upon stimulation [27,28]. Sprouty2 can bind to Grb2 through the SH3 binding motif in the C-terminus [25]. It can also bind to Shp2 phosphatase [29], Raf1 and Tesk1 *via *the cysteine rich domain (CRD) [30,31]. Human Sprouty2 is known to inhibit cell migration and proliferation in response to serum and growth factors [32]. When overexpressed, it is capable of inhibiting anchorage-independent cell growth, cell migration and invasion [33], tumor growth and metastasis [34]. Like most tumor suppressors, the expression of Sprouty is downregulated in many cancers such as breast cancer [35], prostate cancer [36,37], liver cancer [38,39], non-small cell lung cancer [40] and B-cell lymphomas [41] by variable mechanisms depending on the individual cancer type.

Our study indicates that the biochemical status of the cell plays a crucial role in determining its susceptibility to oncogenic transformation. We have identified a novel relationship between the tumor suppressor Sprouty2 and the *Env *oncogene *in vitro*, both signaling through overlapping pathways. Overexpression of Sprouty2 seems to inhibit the oncogenic transformation induced by *Env*, resulting in suppressed tumor formation potential. We found that amino acid Y55 is crucial for Sprouty2 function.

## Results and Discussion

### *Env*-mediated transformation induces Sprouty2 and inhibits cell migration

**Figure 1:**
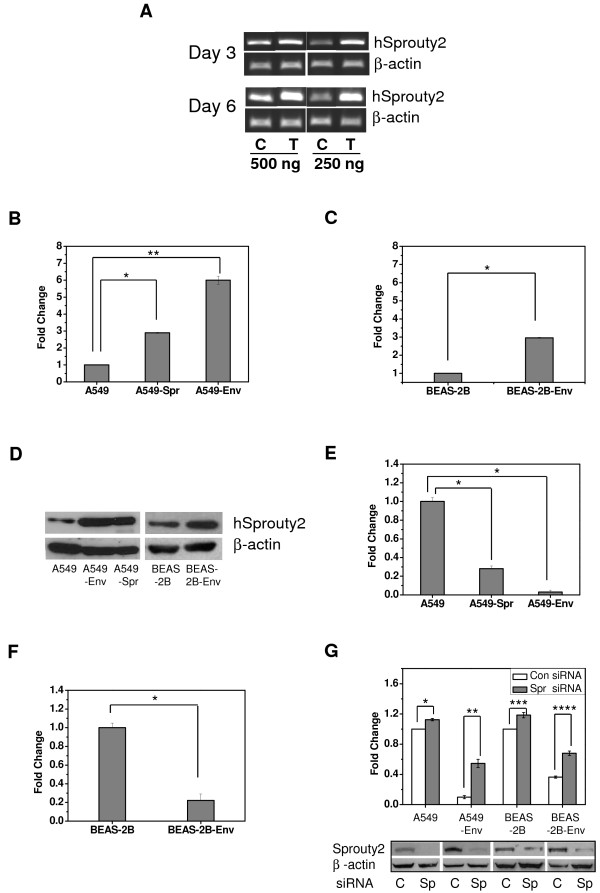
**Induction of Sprouty2 and inhibition of cell migration in JSRV *Env *transformed cells**. (A) A549 cells were transfected with a plasmid carrying JSRV *Env *gene (T) or the empty vector (C), and the expression of Sprouty2 and β-actin was monitored on days 3 and 6 post transfection by RT-PCR using different template concentrations (500 ng and 250 ng). Data represent two independent experiments. (B) and (C) Quantitative RT-PCR analysis depicting the fold increase in Sprouty2 mRNA levels in the stable cell lines A549-Spr and A549-Env compared to A549 (*P < 0.0007, **P < 0.0001) (B) and in BEAS-2B-Env compared to BEAS-2B (C) *P < 0.0001. (D) Western blot analysis of the expression of Sprouty2 protein and β-actin in stably transformed cell lines compared to the parental cell lines. Data represent five independent experiments. (E) and (F) Cell migration assay: Equal number of A549, A549-Spr and A549-Env cells (E) or BEAS-2B and BEAS-2B-Env cells (F) were allowed to migrate across the 8 μm porous membrane in a Boyden chamber in response to serum. After 15 h, the migrated cells were fixed, stained and counted (*P < 0.0001). (G) The effect of 200 pmoles of Sprouty2-specific siRNA on the migration ability of A549, A549-Env, BEAS-2B and BEAS-2B-Env cells compared to control siRNA (*P = 0.014, **P = 0.0169, ***P = 0.034, ****P = 0.0111). Corresponding changes in Sprouty2 protein levels compared to β-actin control are depicted in the Western blot shown below the bar diagram for the respective cell lines. (C) indicates control siRNA and (Sp) indicates Sprouty2 siRNA. Data represent two independent experiments.

To analyze the *in vitro *transformation induced by JSRV *Env *gene in normal as well as the cancer cell lines derived from human lung, the full length *Env *gene from JSRV was cloned in pBluescript vector under control of the CMV promoter and used for transfection of the lung adenocarcinoma cell line A549 and the immortalized lung epithelial cell line BEAS-2B. An analysis of the genes transiently induced by *Env *in A549 cells three and six days post transfection showed upregulation of the tumor suppressor Sprouty2 when analyzed by semi-quantitative RT-PCR using different template concentrations (Figure 1A). This novel phenomenon of an oncogene upregulating a tumor suppressor was not observed in BEAS-2B cells transiently transfected with the *Env *gene (data not shown). Regulation of the expression of Sprouty2 in the cells occurs at multiple levels [42], and therefore the exact reason for increased Sprouty2 expression in A549-Env could not be ascertained. We assumed that the upregulation of Sprouty2 might be a standby effect in the course of modulation of cell signaling network by *Env*. We created stable cell lines overexpressing Sprouty2 as well as stable *Env*-transformed cell lines. The expression level of Sprouty2 mRNA in these cell lines was ascertained by quantitative RT-PCR. The results revealed that A549-Spr had 2.9-fold increased amount of Sprouty2 transcripts while A549-Env had a 6-fold increase in Sprouty2 levels compared to A549 (Figure 1B). On the other hand, BEAS-2B-Env cell line had only 2.9-fold increase in the expression of Sprouty2 mRNA compared to BEAS-2B (Figure 1C). These findings support our theory that Sprouty2 induction has a positive correlation to *Env*-mediated transformation.

In addition to Sprouty2, another candidate tumor suppressor HYAL2, was also found to be induced by *Env*-mediated transformation of A549 cells (data not shown); the tumor suppressor activity of HYAL2 is reported to be inhibited by JSRV *Env *[43]. Although usually oncogenes are known to suppress or inactivate tumor suppressors, some tumor suppressors are known to be recruited for oncogene-induced functions such as DNA damage [44], and some proteins like SnoN are identified to function as an oncogene as well as a tumor suppressor [45].

The functional relationship between the oncogenes and the tumor suppressors is therefore multifaceted, and we went ahead to study the correlation between JSRV *Env *oncogene and Sprouty2 tumor suppressor. An analysis of Sprouty2 protein levels in the stable cell lines revealed a significant upregulation in A549-Spr and A549-Env compared to A549, and in BEAS-2B-Env compared to BEAS-2B (Figure 1D). We deduced that the increased expression of Sprouty2 might have significant physiological ramifications and went ahead to test this hypothesis.

Overexpression of Sprouty2 is known to interfere with cell migration and invasion [33]. So, the migration ability of the cell lines under study was compared by allowing them to migrate across a porous membrane in a Boyden chamber. A549 cells *per se *exhibited high migration potential which was reduced by ~70% in A549-Spr, while A549-Env cells were unable to migrate (Figure 1E). Similarly, BEAS-2B cells had good migration capability whereas BEAS-2B-Env cells exhibited ~80% reduction in their migration potential (Figure 1F). The effect of *Env*-transformation on the migration ability was drastic in A549-Env cells abrogating their migration potential, whereas in BEAS-2B-Env cells, the effect was severe resulting in reduced migration ability. Therefore, we hypothesize that Sprouty2 might have a hand in the compromised migration potential of these cells.

To verify the role of Sprouty2 in the regulation of cell migration, A549, A549-Env, BEAS-2B and BEAS-2B-Env cells were treated with 200 pmoles of siRNA for Sprouty2 or with control siRNA and then allowed to migrate. It was observed that siRNA-mediated inhibition of Sprouty2 expression resulted in a corresponding enhancement in cell motility (Figure 1G). The enhancement was more prominent in A549-Env and BEAS-2B-Env cells that had higher levels of Sprouty2 initially and consequently very low migration potential. Inhibition of Sprouty2 expression in the cells enhanced their migration ability, thereby confirming that Sprouty2 played an inhibitory role in cell migration.

To investigate in detail the physiological consequences brought about by Env and Sprouty2, further investigations were carried out.

### *Env *induces proliferation and colony formation in A549-Env

Proliferation and invasion are two distinct fundamental components occupying opposing ends of a spectrum in malignant cells and are not necessarily displayed by the same cells. Invasion, migration and branching morphogenesis are exclusive characteristics of highly invasive cells while highly proliferative cells are highly tumorigenic and display anchorage-independent growth in soft agar [46]. Anchorage-independent growth is an attribute of oncogenic transformation by *Env *that causes cells to loose contact inhibition resulting in the formation of distinct foci in culture [47]. The cell lines were further investigated for their proliferation potential and anchorage-independent growth.

**Figure 2:**
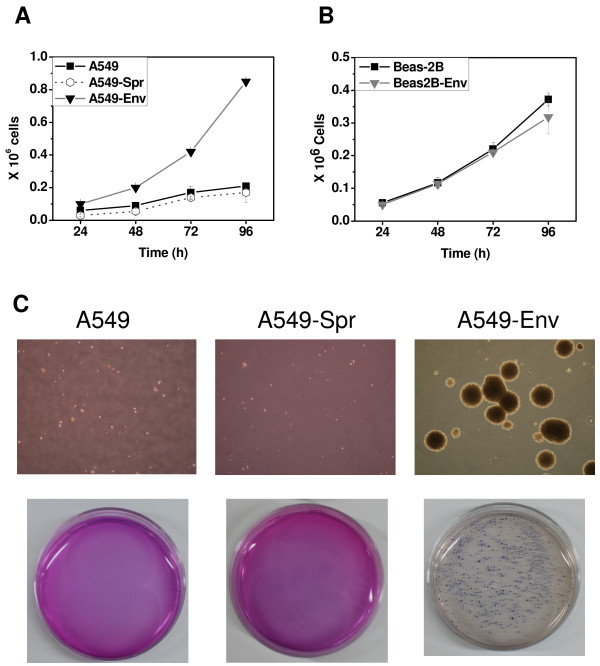
**JSRV *Env *induces proliferation and colony formation in A549-Env cells**. (A and B) Equal number (1 × 10^5 ^cells/well) of A549, A549-Spr and A549-Env cells (A) or BEAS-2B and BEAS-2B-Env cells (B) were allowed to proliferate for four days and live cells were counted every 24 h. (C) A549, A549-Spr and A549-Env cells were cultured in soft agar to assess their anchorage-independent colony formation ability. The colonies were counted and photographed on day 12.

A549-Env had a higher proliferation rate with ~4-fold more cells after 96 hours than A549 and A549-Spr; increased proliferation being a characteristic feature of oncogene-induced transformation (Figure 2A). On the other hand, both BEAS-2B and BEAS-2B-Env had comparable proliferation rates (Figure 2B). Our results clearly demonstrate that the loss of invasive ability induced by JSRV *Env *is distinct from the enhanced proliferation function. *Env*-mediated transformation had converted the highly invasive A549 cells into highly proliferative A549-Env cells. Our results suggest that the choice of invasion versus proliferation and tumor formation functions is more likely to be governed by distinct pathways of signaling, which are probably evoked independently.

In colony formation assay, A549-Env cells formed a number of distinct, large colonies in soft agar in 12 days, a characteristic feature of JSRV *Env*-mediated transformation (Figure 2C). A549-Spr showed inhibition of colony formation, probably due to the inhibitory effect of Sprouty2, as reported earlier [33]. A549, although known to be capable of anchorage-independent growth, could form only very small colonies by day 12, probably owing to its lower proliferation rate compared to A549-Env. It is clear that *Env *had induced higher proliferation rate and colony formation in A549-Env cells in spite of high levels of Sprouty2. Both BEAS-2B and BEAS-2B-Env could not form colonies in soft agar suggesting that *Env *transformation had less effect in the normal epithelial cell line BEAS-2B.

BEAS-2B cells are immortalized human lung epithelial cells that have low transfection efficiency [48], and the reproducibility of transformation assays is reported to be difficult [49]. Therefore it is not surprising that *Env*-mediated transformation of BEAS-2B could induce only limited biochemical and physiological alterations. In an attempt to unravel the underlying mechanisms responsible for *Env*-mediated transformation, an analysis of the status of signaling molecules in these cell lines was carried out.

### *In vivo *tumorigenesis is inhibited by Sprouty2, but enhanced by *Env*

**Figure 3:**
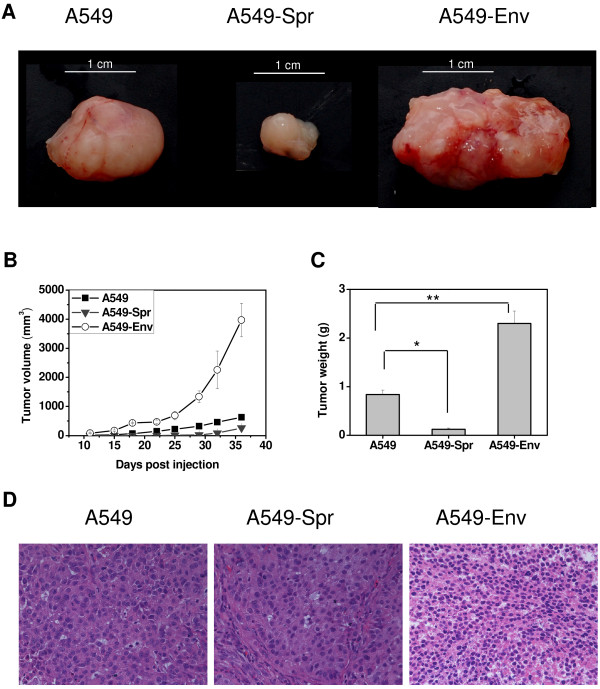
***Env *enhances and Sprouty2 inhibits tumor formation in SCID mice**. (A) Pictures of i*n vivo *tumors formed by A549, A549-Spr and A549-Env cells injected into the subcutaneous tissue of SCID mice (5-7 mice per group) and excised on day 34. (B) The growth rate of tumors formed by the respective cell lines in SCID mice is represented as increase in tumor volume monitored for up to 5 weeks. (C) Average weight of tumors resected from SCID mice 34 days after injection (*P = 0.0003, **P = 0.0006). (D) Hematoxylin and eosin (H&E) staining of sections of xenografts resected from SCID mice to confirm the presence of malignant cells. Magnification 400×.

To investigate the *in vivo *tumor forming potential, A549, A549-Spr, A549-Env, BEAS-2B or BEAS-2B-Env cells were injected subcutaneously into SCID mice and allowed to form tumors. A549 was capable of forming tumors *in vivo *while the tumor forming potential was decreased in A549-Spr that overexpresses the tumor suppressor Sprouty2 (Figure 3A). A549-Env was capable of forming massive tumors, characteristic of oncogenic transformation. All the tumors had pushing margins rather than invading margins at the time of termination of the experiment, and *in vivo *invasiveness was not detected.

The growth rate of tumors as indicated by the progressive increase in tumor volume (Figure 3B) as well as tumor weight (Figure 3C) was the greatest in A549-Env (~2.3 g) and the lowest in A549-Spr (~0.1 g) compared to A549 (~0.8 g) (Figure 3B and 3C). The inhibitory effect of overexpressed Sprouty2 in tumor formation that has been reported earlier [34] is confirmed by our observations.

All the tumors were sectioned and stained with hematoxylin and eosin and the presence of proliferating tumor cells was confirmed (Figure 3D). The sections showed a poorly differentiated adenocarcinoma composed of cells with hyperchromatic nuclei. The tumor formed by A549-Env showed increased cellularity owing to the high proliferation rate of A549-Env cells. BEAS-2B and BEAS-2B-Env failed to form tumors in SCID mice, behaving more like normal epithelial cells without much permanent alterations in their functionality. An analysis of the signaling scenario in these cell types gave an insight into their biochemical attributes.

### Alteration of the signaling network by *Env *and Sprouty2

**Figure 4:**
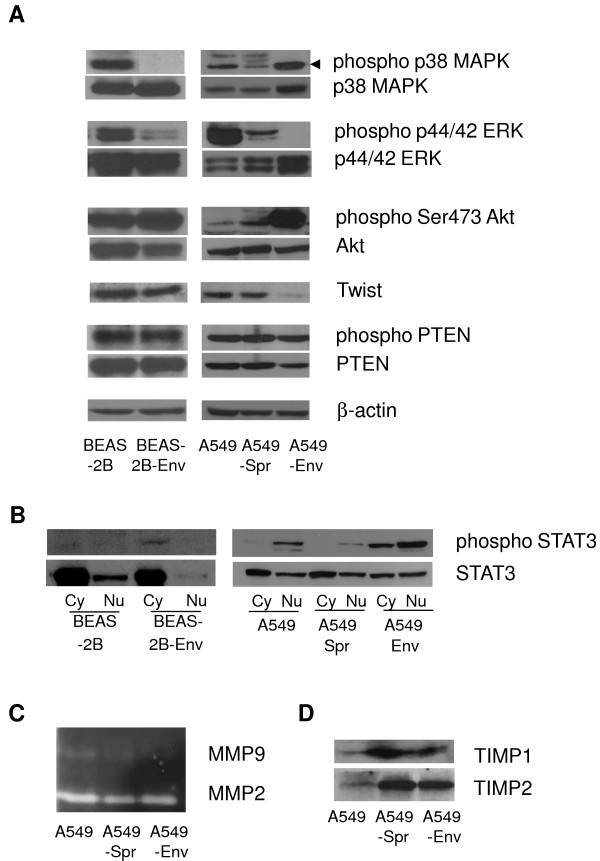
**Alteration of signal transduction by *Env *and Sprouty2**. (A) Cell lysates were prepared by homogenization of A549, A549-Spr, A549-Env, BEAS-2B and BEAS-2B-Env cells. The proteins were separated on 10% acrylamide gel, transferred to nitrocellulose membrane and probed with various antibodies. The results show the levels of phospho p38 MAPK, p38 MAPK, phospho p44/42 ERK, p44/42 ERK, phospho Ser473 Akt, Akt, TWIST, phospho PTEN, PTEN and β-actin in the indicated cell lines. (B) Proteins were extracted from cytoplasmic (cy) and nuclear (nu) fractions of the cells and the levels of phospho STAT3 and STAT3 were ascertained by Western blot. (C) MMP Zymogram: Conditioned media obtained from the cell lines was assayed for gelatinase activity using 10% SDS-PAGE gel to estimate MMP activity. (D) Conditioned media obtained from the cell lines was concentrated; the secreted proteins were separated by SDS-PAGE and immunoblotted with antibodies against TIMP1 and TIMP2.

Characterization of the molecular pathways leading to cancer is a major step towards understanding and combating the disease [50]. The alterations induced by Sprouty2 and *Env *in the signaling scenario of A549 were investigated by Western blot. The mechanism of JSRV *Env*-mediated transformation of cells is not clear and is reported to modulate the PI3K and MAPK pathways [48]. Sprouty proteins are feedback negative regulators of the ERK pathway [51] that is thought to regulate cell invasion [52,53]. Upon analysis, A549 was found to have high levels of phosphor-ERK; A549-Spr had very low levels; and in A549-Env, phosphor-ERK was not detected (Figure 4A). Similarly, BEAS-2B had high levels of phosphor-ERK, which was decreased in BEAS-2B-Env. These observations are consistent with the increased expression of Sprouty2 in the respective transformed cell lines compared to their parental counterparts.

TWIST is a transcription factor that has been detected in various carcinomas and is suggested to enhance the invasive and metastatic ability of cancer cells [54-56]. The expression of TWIST was significantly reduced in A549-Env and BEAS-2B-Env compared to A549 and BEAS-2B respectively (Figure 4A). These observations are consistent with the decreased migration potential of A549-Env and BEAS-2B-Env.

The p38 MAPK pathway is reported to have anti- or pro- proliferative functions depending on the levels of kinase activity and the interplay between all the signaling pathways [57]. Phosphorylation of p38 MAPK was decreased in A549-Spr compared to A549, but was significantly enhanced in A549-Env, probably owing to *Env*-induced signaling. However, in BEAS-2B, the phosphorylation of p38 MAPK was high, which was not seen in BEAS-2B-Env (Figure 4A). The implication of signaling mediated by p38 MAPK in BEAS-2B cells is not clear.

The PI3K/Akt pathway is known to play a crucial role in cell proliferation and survival and shows a high frequency of alterations in cancer [58,59]. Akt phosphorylation was marginally increased in A549-Spr compared to A549. But, A549-Env had very high levels of phosphor-Akt, showing a positive correlation with the observed high proliferation rate (Figure 4A). Similarly, BEAS-2B-Env showed an increase in the phosphorylation of Akt compared to BEAS-2B, consistent with the reported involvement of PI3K/Akt pathway in *Env*-induced transformation [13], although the proliferation rate of all the BEAS-2B cell lines remained similar.

The tumor suppressor phosphatase and tensin homologue (PTEN) is a negative regulator of the PI3K/Akt pathway [60] that can suppress cell growth and tumor formation [61,62]. Active PTEN was lower in A549-Env cells compared to A549 and A549-Spr or to the inactive phosphor-PTEN level (Figure 4A). In BEAS-2B and BEAS-2B-Env, PTEN and phospho PTEN levels were high and comparable, consistent with their inability to form tumors.

The transcription factor STAT3 is hypothesized to play a role in anchorage-independence [63] and cell growth [64]. In A549-Env, high levels of phosphor-STAT3 were detected both in the nucleus and the cytoplasm, indicating over activation, consistent with the increased growth and colony forming potential of the cells (Figure 4B). In A549 that is capable of anchorage-independent growth, phospho STAT3 was detected in the nucleus. But in A549-Spr, BEAS-2B and BEAS-2B-Env, all of which are impaired in anchorage-independent colony formation, phospho STAT3 level in the nuclear fraction was very low (Figure 4B). Increased activation of Akt, STAT3 and reduced expression of PTEN and TWIST are likely to have contributed to the high proliferation and colony formation potential of A549-Env.

Matrix metalloproteinases (MMPs) are enzymes that are involved in the breakdown of the extracellular matrix abetting invasion and metastasis of cancer cells [65]. Tissue inhibitors of metalloproteinases (TIMPs) are inhibitors of MMPs, which decrease cell migration [66]. Usually the MMP/TIMP ratio (MMP2/TIMP2 and MMP9/TIMP1) determines the invasiveness of the cells and is known to be altered in many cancers [67]. To address the role of MMPs and TIMPs in altering the migration ability of the cell lines under study, the activity of MMPs was evaluated by gelatin zymogram and the amount of TIMPs by Western blot. MMP levels were high, and TIMP levels were very low in A549, an invasive cell line (Figure 4C and 4D). In A549-Env, MMP2 level was relatively less, and MMP9 was hardly detectable while TIMP levels remained high, consistent with its reduced migration potential (Figure 4C and 4D). A549-Spr, in spite of having similar levels of MMPs and TIMPs like A549-Env, had less activity in the cell migration assay (Figure 1E). In BEAS-2B and BEAS-2B-Env, the levels of MMPs and TIMPs were low and comparable (data not shown). Elevated TIMPs and decreased MMP9 levels seem to have contributed to the decreased migration efficiency of A549-Spr and A549-Env. Overall, the signaling status of the cells is consistent with the manifested functions in the respective cell lines.

### PI3K/Akt and ERK pathways regulate proliferation and cell migration

The MAPK (p44/42) and Akt pathways have been implicated in JSRV *Env*-mediated transformation in many cell types [8,48]. Since the ERK and PI3K/Akt pathways appear to be involved in cell migration and proliferation respectively in A549 cells, in order to confirm the same, we treated the cells with pharmacological inhibitors of the respective pathways to study their effect on invasion and proliferation.

**Figure 5:**
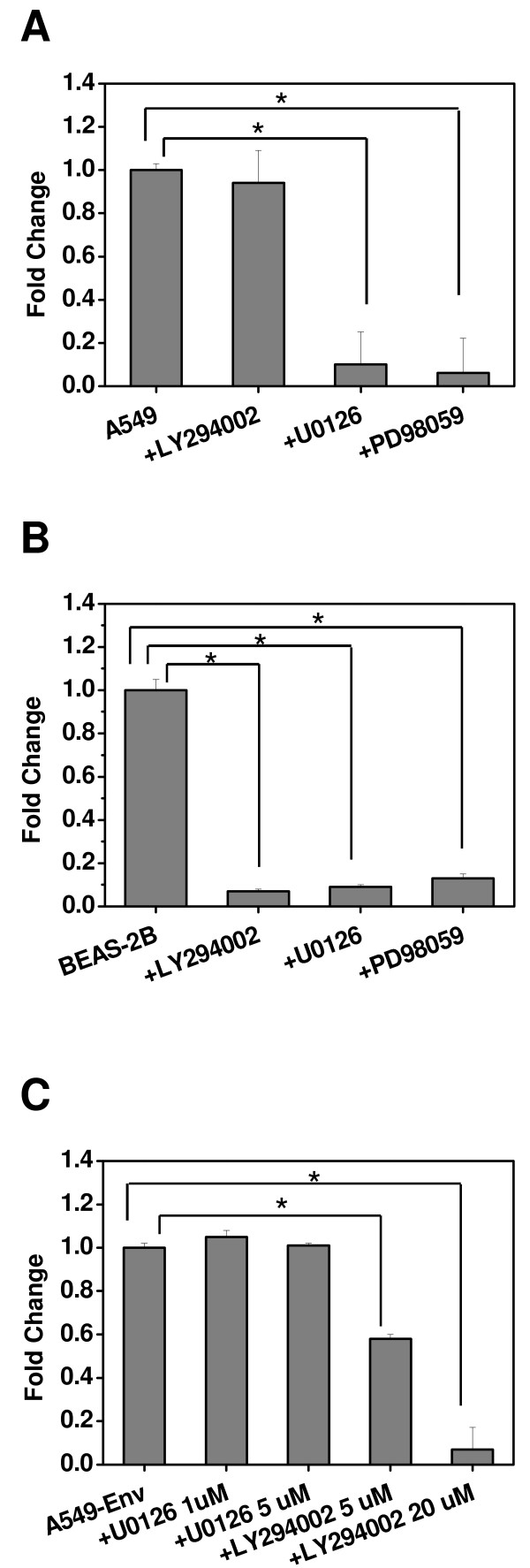
**ERK and PI3K pathways regulate cell migration and proliferation**. A549 (A) or BEAS-2B (B) cells were allowed to migrate across the 8 μm porous membrane in a Boyden chamber either alone or in the presence of PI3K inhibitor LY294002 (20 μM) or MEK inhibitors U0126 (20 μM) or PD98059 (20 μM) in response to serum. After 15 h, the migrated cells were fixed, stained and counted. (A) *P < 0.0001; (B) *P < 0.0003). (C) A549-Env cells were allowed to proliferate either alone or in the presence of MEK inhibitor U0126 (1 μM) or (5 μM) or PI3K inhibitor LY294002 (5 μM) or (20 μM). Data shown represent proliferation at 72 h. (*P < 0.0001).

A549 and BEAS-2B cells are intrinsically invasive and responded differently to the inhibitors of ERK and PI3K. The migration ability of A549 cells was reduced in the presence of either of the MEK inhibitors, U0126 or PD98059 while the PI3K inhibitor LY294002 had no effect (Figure 5A). This observation confirms that the ERK pathway is required for cell migration in A549. This also suggests that the reduced migration ability of A549-Spr and A549-Env might be due to the inhibition of the ERK pathway probably caused by the upregulation of Sprouty2. Inhibition of the p44/42 MAPK pathway by pharmacological inhibitors is known to abolish JSRV *Env*-mediated transformation of cells *in vitro *[8] confirming that this pathway is involved in oncogenic transformation caused by *Env*.

On the other hand, in BEAS-2B cells, the MEK inhibitors as well as the PI3K inhibitor were able to inhibit cell migration (Figure 5B). In BEAS-2B, multiple pathways seem to function in an overlapping manner and therefore a single pathway could not be attributed to a particular physiological function. BEAS-2B-Env cells do not have enhanced proliferation rate and therefore further investigation for attribution of pathway specificity to proliferation was conducted using A549-Env cells.

Akt pathway is highly enhanced in A549-Env cells and therefore is correlated with its very high proliferation potential. When A549-Env cells were allowed to proliferate in the presence of MEK inhibitors or PI3K inhibitor, only the latter (LY294002) was able to inhibit proliferation, confirming that the PI3K/Akt pathway is required for their enhanced proliferation potential (Figure 5C). Our observations suggest that the Akt pathway is involved in proliferation and the ERK pathway in migration of A549 and its derivative cell lines.

### The amino acids Y55 and Y227 are crucial for the function of Sprouty2

Our observations implicate that Sprouty2 has the potential to alter the physiology of A549 and therefore further investigations on the tumor suppressive functions of Sprouty2 were conducted using A549. To ascertain the role of Sprouty2 in inhibiting cell migration, tumor formation and anchorage-independent growth, functional mutants of Sprouty2 were created. Two key tyrosine residues, Y55 and Y227 have been identified in human Sprouty2 protein, mutations of which seem to affect its interaction with the other signaling molecules as well as its function as an ERK inhibitor. Y55 residue is the major tyrosine crucial for the function of Sprouty2, in the absence of which, Y227 can mediate some of its functions [27,68,69]. We created two mutants of Sprouty2 - Y55F and Y227F by site-directed mutagenesis and expressed them in A549 cells to create 'A549-Y55FSpr' and 'A549-Y227FSpr' stable cell lines respectively. The mutants are envisaged to interrupt the functions of endogenous Sprouty2.

**Figure 6:**
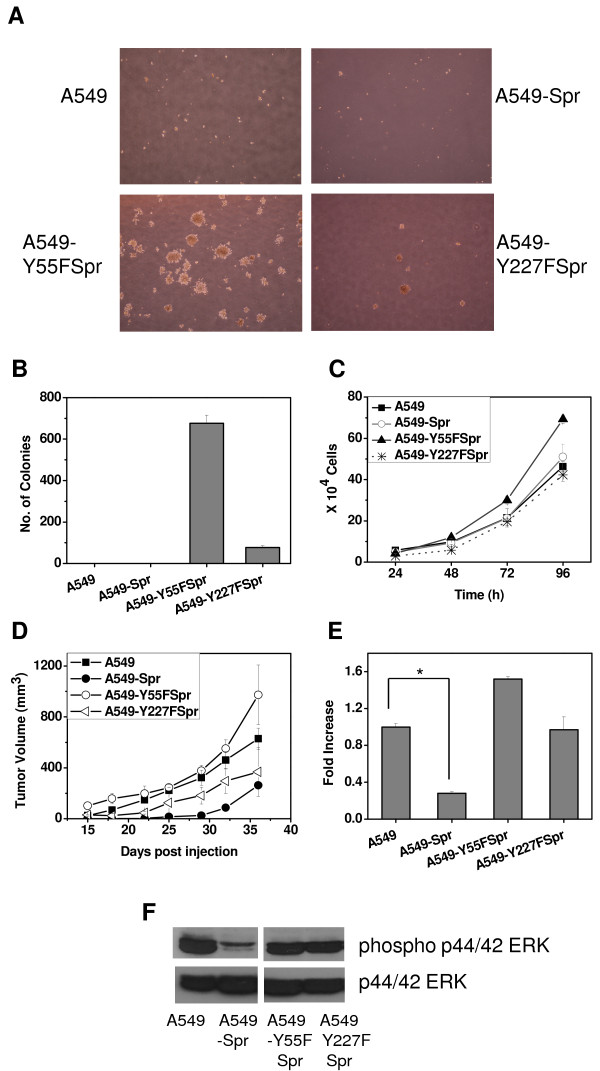
**Tyrosine mutants of Sprouty2 inhibit its tumor suppressive function**. Stable transfectants of A549 over expressing either Y55F or Y227F mutant of Sprouty2 designated A549-Y55FSpr and A549-Y227FSpr respectively were created and assayed for their functional properties. (A) and (B) Colony formation assay: A549, A549-Y55FSpr and A549-Y227FSpr cells were cultured in soft agar to assess their anchorage-independent colony formation ability. (A) The colonies were counted and photographed on day 12. (B) Graphical representation of the number of colonies formed by each cell line. (C) Equal numbers (1 × 10^5 ^cells/well) of A549, A549-Y55FSpr and A549-Y227FSpr cells were allowed to proliferate for four days. Schematic representation of their proliferation rate represented by live cell count every 24 h. (D) *In vivo *tumor formation: A549, A549-Spr or A549-Y55FSpr or A549-Y227FSpr cells were injected into the subcutaneous tissue of SCID mice. Growth rate of tumors formed by the respective cell lines in SCID mice is represented as increase in tumor volume monitored for up to 5 weeks. (E) Migration assay: Cells were allowed to migrate across the 8 μm porous membrane in a Boyden chamber in response to serum. After 15 h, the migrated cells were fixed, stained and counted. (*P = 0.0029). (F) Western blot analysis of the cell lysates of A549, A549-Spr, A549-Y55FSpr and A549-Y227FSpr to check for the levels of phospho ERK p44/42 and total ERK p44/42.

Functional analysis revealed that while both A549-Y55FSpr and A549-Y227FSpr cells were capable of anchorage-independent colony formation, the former was more potent causing an increase in colony size (Figure 6A) as well as colony number compared to A549 (Figure 6B). A549-Y227FSpr formed smaller and fewer colonies than A549-Y55FSpr. The proliferation rate of A549-Y55FSpr was higher than that of A549 while A549-Y227FSpr was comparable to A549 (Figure 6C). These observations corroborate the finding that Y55 is the major tyrosine residue crucial for Sprouty2 function.

When these cells were injected into SCID mice subcutaneously to compare the tumor forming potential, it was observed that the tumor growth rate of A549-Y55FSpr was marginally greater than that of A549, while A549-Y227FSpr had a tumor growth rate less than A549, but greater than A549-Spr (Figure 6D).

The effect of the functional mutants of Sprouty2 on cell migration was investigated. A549-Y55FSpr had ~1.5-fold increased migration potential than A549 while the migration potential of A549-Y227FSpr was comparable to that of A549 (Figure 6E). These observations confirm the inhibitory effect of the tyrosine mutants on endogenous Sprouty2 function and the inhibitory role of Sprouty2 in tumorigenesis, anchorage-independence and migration. These data also confirm that Tyr55 plays a more significant role in Sprouty2 function than Tyr227 and therefore is more effective in disrupting the function of endogenous Sprouty2.

An analysis of the alteration of signaling network in these cell lines revealed that ERK phosphorylation was not inhibited in both A549-Y55FSpr and A549-Y227FSpr, whereas inhibition of ERK phosphorylation is a characteristic feature of A549-Spr (Figure 6F). The profile of other signaling molecules such as Akt, p38 MAPK, STAT3, and PTEN in A549 transfected with the mutants was similar to that of A549 (data not shown). Based on these observations we assume that the major inhibitory effect of wild type Sprouty2 is due to its inhibition of the ERK pathway.

### Overexpression of Sprouty2 makes cells resistant to *Env*-mediated transformation

**Figure 7:**
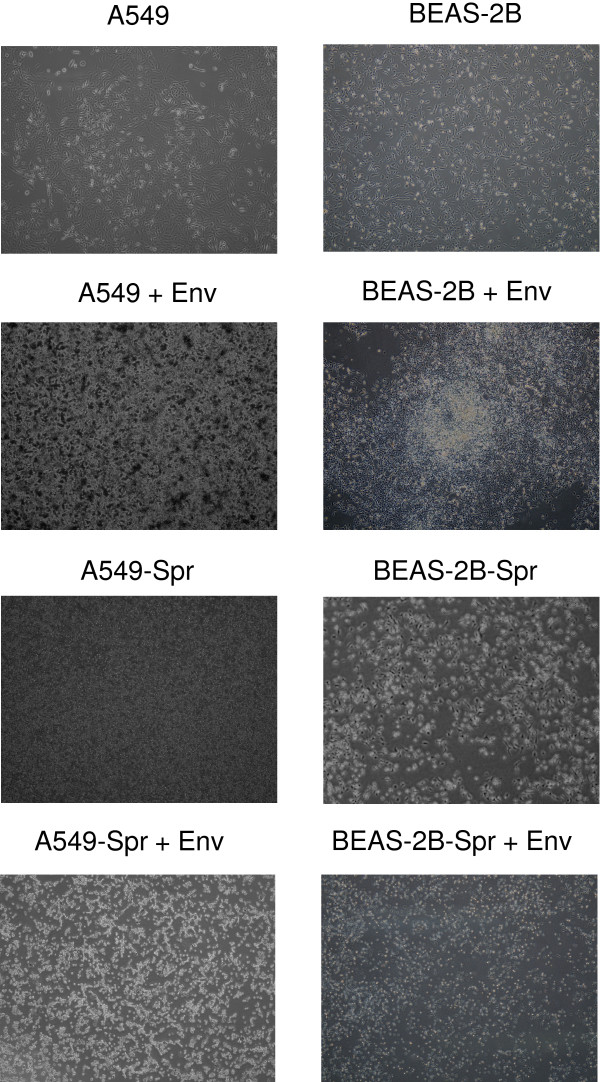
**Sprouty2 overexpressing cells are resistant to *Env*-mediated transformation**. A549, A549-Spr, BEAS-2B and BEAS-2B-Spr cells were transfected with the plasmid carrying *Env *gene by standard calcium chloride method. Foci formation after 14 days was monitored and photographed at 10× magnification.

To study the correlation between Sprouty2 and the viral oncogene *Env*, A549-Spr and BEAS-2B-Spr cells overexpressing Sprouty2 were transfected with a plasmid carrying *Env *gene to allow the formation of distinct foci, a hall mark of *Env*-induced transformation. Fourteen days after transformation with Env, A549 cells showed a number of large distinct foci (A549 + Env) while very few small foci were seen in A549-Spr (A549-Spr + Env) (Figure 7). Similarly, BEAS-2B developed distinct foci upon transformation with *Env *(BEAS-2B + Env) while in BEAS-2B-Spr (BEAS-2B-Spr + Env), foci formation was not observed (Figure 7). Env and Sprouty2 both seem to affect transformation of target cells, with Env promoting it and Sprouty2 antagonizing it. BEAS-2B-Spr had decreased migration rate and decreased phosphor- ERK levels compared to BEAS-2B (data not shown), but otherwise, both the cell lines were comparable in terms of their functionality and the status of signaling molecules. Interference of foci formation in BEAS-2B-Spr and A549-Spr cells indicates that Sprouty2 inhibits *Env*-mediated transformation.

**Figure 8:**
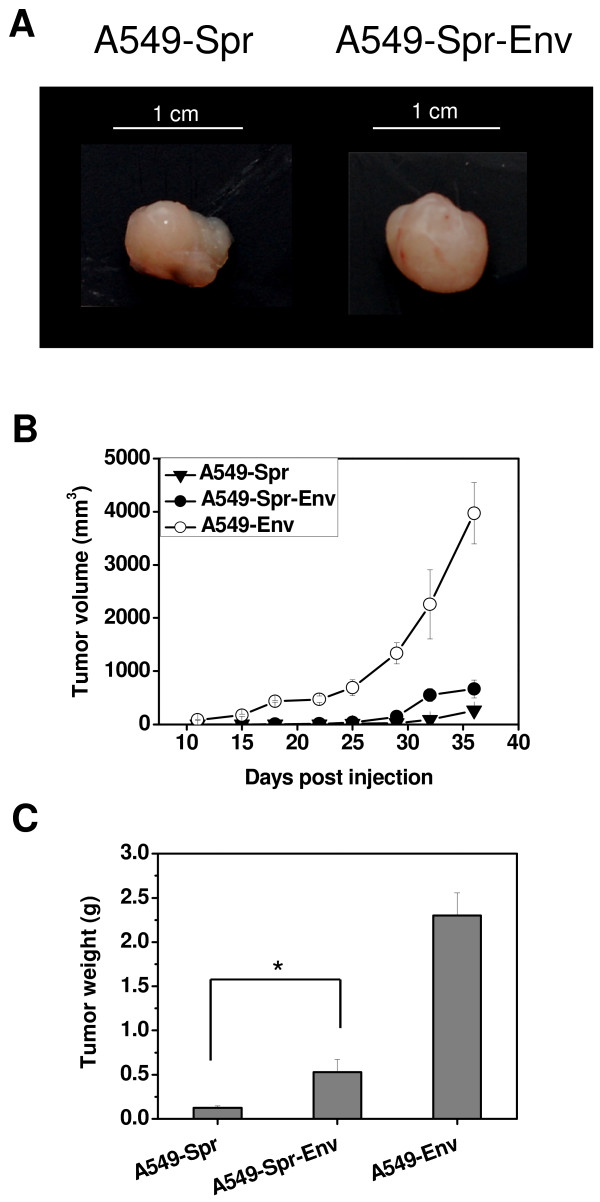
**A549-Spr-Env cells lack enhanced tumor forming potential *in vivo***. (A) Pictures of i*n vivo *tumors formed by A549-Spr and A549-Spr-Env cells injected into the subcutaneous tissue of SCID mice and excised on day 34. (B) Growth rates of the tumors formed by the respective cell lines in SCID mice represented as tumor volume monitored for up to 5 weeks. (C) Average weight of tumors resected from SCID mice 34 days after injection. (*P = 0.0369).

A549-Spr cells transfected with *Env *(A549-Spr-Env) had similar rates of proliferation and migration like A549-Spr and were unable to form colonies in soft agar (data not shown). When injected into SCID mice, their tumor forming potential was only marginally enhanced than that of A549-Spr in terms of tumor size (Figure 8A and 8B) and tumor weight (Figure 8C). *Env *was therefore unable to endow rapid proliferation and tumor formation potential to A549-Spr cells. These results indicate that overexpression of Sprouty2 in both A549 and BEAS-2B cells that are normally susceptible to *Env*-mediated transformation, had made them resistant to the same. This can be attributed to the overexpression of the tumor suppressor Sprouty2 and subsequent alterations in the physiological and signaling status of the cells.

**Figure 9:**
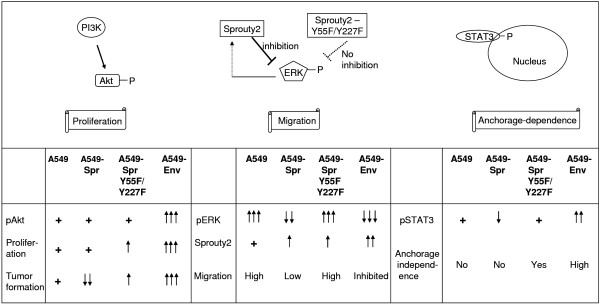
**Schematic diagram representing the alteration of signaling network by Sprouty2 and Env**. Schematic diagram represents the interaction of signaling molecules and their proposed physiological outcomes. The levels of phospho Akt, phospho ERK, Sprouty2 and phospho STAT3 in the cell lines A549, A549-Spr, A549-Y55FSpr/A549-Y227FSpr and A549-Env are represented. ↑ indicates upregulation, ↓ indicates downregulation, + indicates presence without any increase or decrease. The functional outcomes in terms of proliferation, tumor formation, migration and anchorage-independent colony formation in the above mentioned cell lines are also represented.

Oncogenesis results from changes in kinetics or abundance of proteins in signal transduction networks with the control dispersed over many components. While the MAPK and PI3K pathways are crucial for *Env *to induce transformation and proliferation, Sprouty2 also has some connections to these pathways. The effect of Sprouty2 (wild type as well as Y55F or Y227F mutants) and *Env *on the major signaling elements and their effect on the functional outcomes of different cells are depicted in Figure 9. Sprouty proteins are well documented to be feedback negative regulators of the MAPK pathway [20,21]. Sprouty2 is reported to bind to phosphatidylinositol-4, 5-biphosphate, a substrate for PI3K by means of its translocation domain [70]. Mouse Sprouty4 is reported to have an inhibitory effect on Akt phosphorylation [71]. Therefore, resistance to *Env *by modulation of PI3K pathway by Sprouty2 is a possibility and can not be ruled out. We could not identify any direct interaction between Env and Sprouty2 proteins (data not shown), as has been documented for many oncoprotein-tumor suppressor protein pairs. Multiple oncoproteins and tumor suppressor proteins have been found to act through the same signaling pathway, to cause or prevent cellular transformation [72]. Similarly, Env and Sprouty2 might affect the same signaling pathways in either a synergistic or antagonistic manner. Parallel Ras/MAPK and PI3K pathways with common connections [73,74] are known to exist in many scenarios. We therefore propose dual regulation of the PI3K/Akt and ERK pathways by both Env and Sprouty2, thereby constituting a functional cross talk. We propose that Sprouty2 resists *Env*-mediated transformation by modulating the signaling pathways, subsequently altering the biochemical status of the cells to make them resistant to oncogenic transformation.

## Conclusions

Proliferation and invasion functions can be governed by distinct signaling pathways in the cells and therefore can be evoked independently in the target cells. Oncogenic *Env *from JSRV and the tumor suppressor human Sprouty2 participate in overlapping signal transduction pathways and therefore are capable of influencing each other, determining the susceptibility of target cells to oncogenic transformation. Both play very relevant roles in cancer induction, progression and invasion. Sprouty2 has a clear role in cell migration, invasion and tumor formation, and its Y55 residue plays a crucial role in its functionality. Sprouty2 shows distinct potential for being exploited as an anti-cancer therapeutic agent for tumor regression and inhibition of cancer invasion and metastasis.

## Methods

### Cell culture

A549, lung adenocarcinoma cell line and its transformants were maintained in Dulbecco's modified Eagle's medium with high glucose (4.5 g/L) supplemented with 10% bovine serum, 2 mM L-glutamine, 100 units/ml penicillin and 100 units/ml streptomycin (Invitrogen, CA, USA) in a 5% CO_2 _humidified incubator at 37°C. Both stable and transient transfections were done by standard calcium chloride method, unless otherwise indicated. Cells were grown to 80% confluency in a 10-cm dish and were transfected with the plasmids carrying *Sprouty *or JSRV *Env *genes. In short, 28 μg of plasmid DNA was mixed with 86.8 μl of 2 M CaCl_2 _solution and the volume was adjusted to 600 μl with sterile distilled water. This solution was added dropwise with constant stirring to equal volume of HEPES buffered saline (0.28 M NaCl, 0.05 M HEPES, 1.5 mM Na_2_HPO_4_, pH 7.0) and the resultant suspension was added to the cells and incubated overnight. Fresh medium was replaced in the morning. A549 and BEAS-2B cells were stably transformed with pCDNA 3.1(-)-Sprouty2 to create A549-Spr and BEAS-2B-Spr cell lines that overexpressed Sprouty2. A549 was transfected with Sprouty2 mutants to create A549-Y55FSpr and A549-Y227FSpr cell lines. A549 and BEAS-2B cells were transfected with pBS-Env and the stable clones were selected from the foci of transformed cells, and developed into A549-Env and BEAS-2B-Env cell lines. *Env*-transformed cells were selected based on their foci forming ability and serum independence as described previously [9,75]. Wild type or mutant Sprouty-transformed cells were selected with 600 μg/ml of G418 (Sigma-Aldrich, St Louis, MO, USA). BEAS-2B, lung epithelial cell line was maintained in LHC-9 medium (Invitrogen) supplemented with 100 units/ml penicillin and 100 units/ml streptomycin and maintained as above.

### Plasmids and primers

Full length of JSRV Envelope gene [Genbank:AF105220] (nucleotides 5348 to 7195) cloned in pBluescript (KS^-^) vector under CMV promoter was gifted by Hung Fan, University of California, Irvine, USA. Full length cDNA of human Sprouty2 gene was cloned by RT-PCR from A549 cell line using the following primers, forward: GGGAATTCCCAAGACCTGATGGAGGCCAG and reverse: CAGGGATCCCTATGTTGGTTTTTCAAAGTTCCTAG as described previously [33] into the expression vector pCDNA3.1(-) using EcoR1 and BamH1 restriction enzymes. The PCR conditions were: 45°C for 30 min, 94°C for 5 min; followed by 35 cycles of 94°C for 45 sec, 59°C for 45 sec and 72°C for 75 sec; followed by 72°C for 7 min. The dominant negative mutants of Sprouty2, Y55F and Y227F were created by site-directed mutagenesis by PCR using Pfu DNA polymerase and the following primers. Y55F forward: CCGAAACACCAATGAGTTCACAGAGG, Y55F reverse: CCTCTGTGAACTCATTGGTGTTTCGG, Y227F forward: GGTCTCTTCTTT CACTGTTCTAATG and Y227F reverse: CATTAGAACAGTGAAAGAAGAGACC. PCR conditions for creation of Y55F mutant were: 94°C for 5 min; followed by 30 cycles of 94°C for 45 sec, 55°C for 45 sec and 72°C for 13 min; followed by 72°C for 30 min; and for Y227F mutant, 94°C for 5 min; followed by 30 cycles of 94°C for 45 sec, 52°C for 45 sec and 72°C for 13 min; followed by 72°C for 30 min. After the PCR reaction, Dpn1 restriction enzyme was used to digest the template DNA. All the constructs were confirmed by restriction enzyme digestion and sequence analysis. All the enzymes were purchased from New England Biolabs (Ipswich, MA, USA).

### RT-PCR

RNA samples were isolated from A549 cells transiently transfected with the empty vector (pBS) or *Env *gene cloned in pBS (pBS-*Env*). On day 3 and 6, the cell lines were treated with TRIZOL reagent (Invitrogen) and total RNA was isolated following the manufacturer's instructions. Human Sprouty2 mRNA and β-actin mRNA were detected by reverse transcription-polymerase chain reaction (RT-PCR) analysis using one-step RT-PCR Premix reagent (iNtRON Biotechnology, Korea) and the following primers: Sprouty2 forward: 5'-CCAAGACCTGATGGAGGCCAG-3', Sprouty2 reverse: 5'-TGTTGGTTTTTCAAAGTTCCTAGG-3', β-actin forward: 5'-TGCGTGACATTAAGG AGAAG-3' and β-actin reverse: 5'-CTGCATCCTGTCGGCAATG-3'. The PCR conditions were 45°C for 30 min, 94°C for 5 min; followed by 35 cycles of 94°C for 45 sec, 59°C (for Sprouty) or 56°C (for β-actin) for 45 sec and 72°C for 75 sec, followed by incubation at 72°C for 7 min.

### Quantitative RT-PCR

Total RNA (2 μg) isolated from the stable cell lines A549, A549-Spr and A549-Env was converted to cDNA using Superscript II reverse transcriptase enzyme and oligo dT primers (Invitrogen) following the manufacturer's instructions. The expression levels of hSprouty2 relative to the house keeping gene, glucose 6-phosphate dehydrogenase (G6PD) was assessed in the stable cell lines by quantitative PCR analysis of the cDNA using ABI PRISM 7900 sequence detection system using SYBR Green with the following primers: Sprouty forward: TGGC AAGTGCAAATGTAAGG and Sprouty reverse: CTTGTCGCAGATCCAGTCC, G6PD forward: GCAAACAGAGTGAGCCCTTC and G6PD reverse: GGCCAGCCACATAG GAGTT. Data were expressed using the comparative C_t _method [76] as fold increase in hSprouty2 expression compared to the internal control, G6PD.

### Western Blot Analyses

Western blot was carried out as described [16] using the SuperSignal West Pico chemiluminescent substrate (Pierce, Rockford, IL, USA) with the following antibodies against phospho Thr180/Tyr182 p38 MAPK, p38 MAPK, phospho Thr202/Tyr204 p44/42 ERK, p44/42 ERK, phospho Ser473 Akt, Akt, phospho Tyr705 STAT3, STAT3, phospho Ser380 PTEN, PTEN (Cell Signaling Technology, MA, USA), Sprouty (Upstate Biotechnology, Billerica, MA, USA), TWIST (Santa Cruz Biotechnology, CA, USA), TIMP1, TIMP2, β-actin (Abcam, Cambridge, MA, USA) followed by the appropriate secondary antibodies conjugated to HRP (Thermo Fisher Scientific, MA, USA). Cytoplasmic and nuclear extracts were prepared using NE-PER kit (Pierce) following manufacturer's instructions. For detection of secreted TIMPs, conditioned media (without FBS) obtained from the different cell lines were concentrated 20-fold using Amicon Ultra centrifugal filter devices (10 Kea) (Millipore, MA, USA) and sample corresponding to 1 ml medium was loaded per well. The blots were quantified using Image J software (NIH, USA).

### MMP Zymogram

20 μl of conditioned media (without FCS) obtained from different cell lines after 24 h incubation were assayed for gelatinase activity using 10% SDS-PAGE gels containing gelatin (0.1% w/v; Sigma-Aldrich) [77,78]. Gels were stained with 0.2% Coomassie brilliant blue R-250 (Bio-Rad Laboratories, CA, USA) and destained in a solution containing 30% methanol and 10% acetic acid. Gelatinase activity was visualized as cleared regions in the blue gels.

### Proliferation assay

Cells were serum starved over night and seeded (1 × 10^5 ^cells per well) in a 24- well culture plate in triplicates in DMEM medium with 10% FBS and incubated at 37°C in a 5% CO_2 _humidified incubator. After 24, 48, 72 and 96 hours, the live cell number was determined by trypan blue exclusion using a haemocytometer. When using pharmacological inhibitors, the cells were pretreated with MEK inhibitors U0126 (5 or 20 μM) or PI3K inhibitor LY294002 (5 or 20 μM) (Sigma-Aldrich) for 30 min and allowed to migrate in the presence of the inhibitors with periodical addition every 36 h.

### Migration assay

*In vitro *migration assays were performed using Corning Costar transwell supports (Corning, NY, USA) containing a gelatin-coated polycarbonate membrane filter (6.5 mm diameter, 8 μM pore size) in a 24-well assay system. DMEM with 10% FBS was placed in the lower chamber and in the upper chamber 50,000 cells suspended in DMEM with 1% FBS were placed. The setup was kept in 5% CO2 humidified incubator. After 15 h, the migrated cells in the lower surface were fixed with 4% formaldehyde, stained with crystal violet, viewed under a microscope, photographed and counted.

When using pharmacological inhibitors, the cells were pretreated with the MEK inhibitors PD98059 (20 μM) or U0126 (20 μM) or the PI3K inhibitor LY294002 (20 μM) for 30 min and then allowed to migrate in the presence of the inhibitors.

### siRNA to Sprouty2

Cells were grown in a 6-well plate to 80% confluency and transfected with 200 pico moles of human Sprouty2-specific siRNA or control siRNA (Santa Cruz Biotechnology). To 50 ĀL of culture medium pre-mixed with 1 ĀL of TurboFect transfection reagent (Fermentas, Maryland, USA), 200 pmoles of siRNA was added and the mixture was added per well of the 6-well plate. Cells were incubated at 37°C for 24 h and then trypsinized, counted and subjected to cell migration assay or cell lysates were prepared for Western blotting as described above.

### Colony formation assay

In 60 mm culture dishes, 0.5% agarose in DMEM with 10% FBS was added as the base agar followed by a top layer containing 5000 cells in DMEM with 0.35% low melting agarose and 10% FBS. DMEM with 10% FBS was overlaid on the top agar. Plates were incubated in 5% CO_2 _humidified incubator for 14 days with periodical medium changes. The colonies were stained with crystal violet and counted. Triplicates were maintained for each group.

### Tumor cells xenografts in SCID mice

Subcutaneous tumors were generated by injecting 1 × 10^6 ^cells in 0.1 ml saline subcutaneously in the thighs of 7 weeks old female NOD.CB17-Prkdc^scid ^(SCID) mice purchased from the National Experimental Animal Center (Taipei, Taiwan ROC). Five to seven mice were used for each treatment and they were monitored twice weekly for tumor development, and tumor diameter was measured using calipers. Tumor volume was determined using the formula, volume = 0.52 × (width) × (length). The tumors were resected after 34 days, weighed and processed for histological analysis. All the experimental procedures were in accordance with the institutional guidelines of Animal Care and Use Committee of the Animal Facility in National Health Research Institutes.

### Histological analysis

Excised tumors were fixed in 10% buffered neutral formalin solution (Sigma-Aldrich) overnight, dehydrated, embedded in paraffin (Thermo Fisher Scientific), sectioned at 4 μm thickness and stained with 1% hematoxylin and eosin solution (Sigma-Aldrich). Bright field microscopy pictures were taken at 400× magnifications.

### Statistics

All the experiments were repeated independently 2-5 times.

Data are presented as mean ± SE. Differences between the groups were assessed by paired Student's *t *test using Graphpad software.

## Abbreviations

JSRV: Jaagsiekte sheep retrovirus; Env: Envelope; PI3K: phosphatidyl inositol 3-kinase; ERK: extracellular signal regulated kinase; STAT3: signal transducer of activator of transcription 3; PTEN: phosphatase and tensin homologue; MAPK: mitogen-activated protein kinase; MMP: matrix metalloproteinase; SCID: severe combined immunodeficiency; TIMP: tissue inhibitor of metalloproteinase; HYAL2: hyaluronidase 2; MEK: mitogen-activated protein kinase/extracellular signal-regulated kinase

## Competing interests

The authors declare that they have no competing interests.

## Authors' contributions

EC was involved in the conception and design of experiments, acquisition, analysis and interpretation of data and drafting of the manuscript. YWL and FD were involved in the design and execution of some experiments. YHC supervised the project, analysis and interpretation of data, and commented on the manuscript. All authors read and approved the final manuscript.
